# Anomalous evolution of broadband optical absorption reveals dynamic solid state reorganization during eumelanin build-up in thin films

**DOI:** 10.1038/s41598-017-00597-8

**Published:** 2017-03-31

**Authors:** Carmela Bonavolontà, Corrado de Lisio, Marco d’Ischia, Pasqualino Maddalena, Paola Manini, Alessandro Pezzella, Massimo Valentino

**Affiliations:** 1 Department of Physics “E. Pancini”, University of Naples “Federico II”, Via Cintia, I-80126 Napoli, Italy; 2 INFN, Sezione di Napoli, Via Cintia, 80126 Napoli, Italy; 3 CNR-SPIN U.O.S. di Napoli, Via Cintia, 80126 Napoli, Italy; 4 Department of Chemical Sciences, University of Naples “Federico II”, Via Cintia 4, I-80126 Napoli, Italy; 5 Institute for Polymers, Composites and Biomaterials (IPCB), CNR, Via Campi Flegrei 34, 80078 Pozzuoli (Na), Italy

## Abstract

The origin of eumelanin optical properties remains a formidable conundrum preventing a detailed understanding of the complex photo-protective role of these widespread natural pigments and the rational design of innovative bioinspired materials for optoelectronic applications. Here we report the unusual kinetic and thickness-dependent evolution of the optical properties of black eumelanin polymers generated by spontaneous aerial polymerization of 5,6-dihydroxyindole (DHI) thin films (0.1–1 μm), consistent with peculiar solid state reorganization mechanisms governing broadband absorption. The complete reversal of eumelanin UV-visible transmittance spectrum curvature on passing from 0.2 to 0.5 μm thick films, the marked increase in visible extinction coefficients with increasing film thickness and the higher UV extinction coefficients in slowly vs. rapidly generated polymers concur to support distinct dynamic regimes of solid-state molecular reorganization at the nanoscale level and to do affect the development of broadband visible absorption. Solid state control of molecular reorganization disclosed herein may delineate new rational strategies for tuning optical properties in eumelanin thin films for optoelectronic applications.

## Introduction

The deposition of black insoluble polymers by oxidative polymerization of 5,6-dihydroxyindole (DHI) is a chemically complex, yet still poorly understood process that mimics the biosynthesis of eumelanins^1^ in man and mammals and that provides a most useful model system to inquire into the structure and physicochemical properties of these functional pigments^1, 2^. Over the past decade, interest in this process has gradually shifted from its biological relevance in relation to the structure, biosynthesis and photo-protective properties of human pigments, to its possible exploitation for the development of soft, biocompatible, bioavailable and biodegradable eumelanin-like materials for optical, optoelectronic and bio-electronic applications^3^. The expectedly enormous technological opportunities offered by eumelanins stem largely from their peculiar set of physicochemical properties, making them unique among bioinspired functional materials. Eumelanins exhibit acidic, basic and chelating groups optimally suited to interact with cells; absorb throughout the entire UV-visible range and can sustain both ionic and electronic currents following visible-light-induced stimulation; display efficient and tunable free radical properties, redox activity, and aggregation dependent behavior; can be tailor-made from a variety of monomer precursors; can respond dynamically to chemical stimuli and physical signals by altering their properties; and can form highly adhesive thin films and interfaces for biomedical applications compatible with sterilization protocols.

Unfortunately, despite considerable advances in the past few years, progress toward a mature eumelanin-based technology is partly hindered by a number of gaps and issues that include the lack of detailed structure-property-function relationships and, especially, the marked difficulties encountered when attempting at controlling their multiple levels of chemical and physical disorder to enhance, optimize or tailor functionality^4^. Until a few years ago, moreover, a virtually insurmountable technological issue, due to the complete insolubility of eumelanins in all solvents, was the preparation of smooth and homogeneous device-quality films. Several approaches have been described in the literature, including use of ammonia solution as solvent^5^, electrochemical deposition^6^ or laser deposition^7^. In most cases, however, polymer deposition is made possible only by solubilization caused by extensive structural degradation and a profound alteration of the original structural and physical properties, accompanied by increased structural heterogeneity.

Recently, an expedient protocol to overcome solubility issues in eumelanin film deposition was reported^8^ which involved spin coating deposition of homogeneous thin films of freely soluble colorless DHI followed by exposure to gaseous ammonia in the presence of air (ammonia-induced solid state polymerization, AISSP). Under AISSP conditions, DHI undergoes quantitative polymerization to eumelanin in about 24 h without significant structural degradation and/or morphological alteration.

Besides opening novel technological perspectives in material science^9, 10^, the AISSP methodology has paved the way to unprecedented insights into the aggregation-controlled mechanisms of visible light absorption in the solid state, an issue of the utmost importance for optical and optoelectronic applications.

Herein, we report for the first time a detailed investigation of the evolution of the optical properties of eumelanin growing *in the solid state* by expedient use of a variant of the AISSP methodology for eumelanin film synthesis. This methodology, hereafter referred to as spontaneous solid state polymerization (SSSP), was based on the omission of the ammonia treatment step to achieve DHI conversion under the mildest possible conditions and at very slow rates. Availability of the SSSP procedure was expected to yield unprecedented insights into the role of *solid state conditions* in determining the broadband absorption properties of eumelanins, integrating the results of other studies carried out on different eumelanin-like systems, e.g. polydopamine films^11^. Specific aims of the study were to address the role of polymerization kinetics and film thickness on the visible absorption properties of the final synthetic eumelanin against similar thickness-matched films produced by the previous AISSP protocol. Although both substrate and reaction conditions reported in this paper are mainly of technological relevance, they nonetheless provide the simplest model possible to elucidate the origin of the band broadening process in support of more biologically oriented studies on melanosome absorption properties^12–16^. Direct investigation of the optical properties of natural eumelanins in melanosomes is a difficult task due to the insolubility and heterogeneous character of the pigment. To this aim, model bottom-up studies of polymer formation in the solid state appear a useful tool, since they focus on the core mechanisms of chromophore development as separated from the effects of the biological environment of eumelanin assembly, including the natural complexity of the enzymatic pathway from tyrosine, the participation of proteins and other cellular ingredients, as well as inorganic components and hydration.

## Results

**Figure 1:**
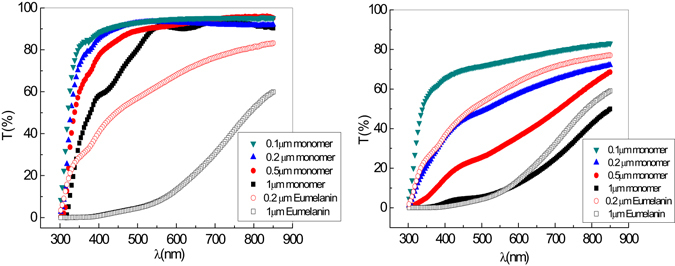
Transmittance spectrum of DHI films soon after deposition (left, t = 0) and after aging (right, t = 4 weeks). For comparison, the transmittance spectra of two separate eumelanin thin films (0.2 μm and 1 μm) prepared by the AISSP procedure are also reported in each graph. The absorption profiles are reported in Figure S1.

Figure 1 shows the transmittance changes in the visible region following SSSP in DHI thin films of various thickness.

The data show a marked decrease in transmittance of DHI thin films after a period of 4 weeks. Complete conversion of DHI to eumelanin-type materials was supported by the substantial superposition (right panel) of the transmittance curves of the 0.2 μm (blue triangles) and 1 μm (black squares) samples produced by SSSP with those of matched AISSP-generated 0.2 μm eumelanin films (open red circles) and 1 μm eumelanin (open black squares), respectively.

Interestingly, although transmittance spectra of 0.1 μm and 0.2 μm films were found to simply translate vertically after 3 weeks, virtually retaining their original curve shape, in the case of 0.5 μm and 1 μm film samples, the oxidation process was characterized by a consistent change in curvature. This change was due to a more significant decrease in transmittance in the visible wavelength range, i.e. 400–600 nm, relative to shorter wavelengths, e.g. 300 nm.

To confirm the role of oxygen, an accelerated aging experiment was carried out in N_2_ atmosphere and air (see SI Figure S5) by thermal treatment of the films at 60 °C for 90 min. Data disclosed the critical role of oxygen in promoting the DHI polymerization and the stability of the film under oxygen free atmosphere.

**Figure 2:**
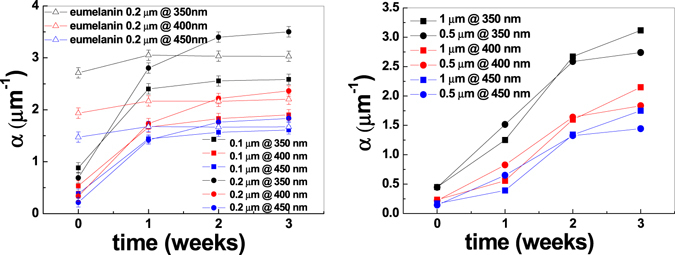
Absorption coefficient of the films as a function of aging time at different wavelengths, namely 350 nm, 400 nm and 450 nm: (left) DHI thin films of 0.1 μm and 0.2 μm undergoing SSSP, and the 0.2 μm DHI films subjected to AISSP; (right) DHI thin films of 0.5 μm and 1 μm.

Further insights into eumelanin optical properties and their dependence on thickness were obtained by plotting the absorption coefficient, α, of 0.1 μm and 0.2 μm DHI films undergoing SSSP as a function of time at three different wavelengths (350, 400 and 450 nm) (Fig. 2). The same information for 0.2 μm eumelan in thin film produced by AISSP is also included.

Some important remarks can be deduced from these plots. First, air exposed eumelanin films produced by the AISSP methodology do not display significant absorption changes with time, denoting complete polymerization of DHI and elevated redoxstability of the final polymer. A wavelength-dependent increase in the absorption coefficients of eumelanin with thickness is also noticeable, with the maximum difference occurring at 350 nm. Second, the thinner the film, the faster the rate of absorption development and the earlier the attainment of a plateau. Third, and most important, eumelanin films produced by SSSP at slower rates exhibit higher absorption coefficients than thickness-matched films produced by AISSP.

The variation of the absorption coefficient, Δα = α_3 week_ − α_0 week_, is summarized in Table 1 for 0.1 μm (Δα_0.1_) and 0.2 μm (Δα_0.2_) thick films, confirming the marked wavelength-dependent increase in the absorption coefficients with thickness. These data are consistent with an influence of the solid state conditions on the synthetic process and developing of the eumelanin absorption profile.

**Table 1:** Absorption coefficient variation at wavelength of 350 nm, 400 nm and 450 nm.

λ (nm)	Δα_0.1_ (µm^−1^)	Δα_0.2_ (µm^−1^)	Δα_0.2_/Δα_0.1_
350	1.70	2.81	1.65
400	1.37	2.03	1.48
450	1.23	1.62	1.32

The observation of the slow variation of the optical absorption as a function of the wavelength suggests that melanins behave as amorphous materials^17^. According to the Tauc model, the optical absorption as a function of the wavelength in amorphous materials generally follows a relation of the type (αhν)^1/2^ ∝ (hν − E) where α is the absorption coefficient, hν is the energy of the photons with which the sample is illuminated, and E defines the optical gap.

**Figure 3:**
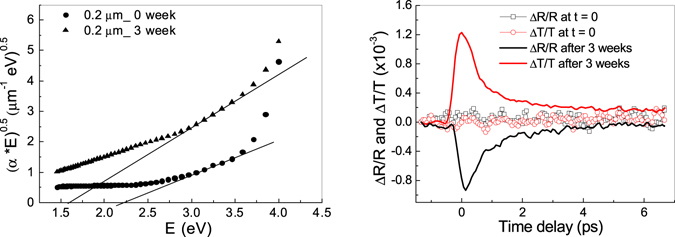
(left) Tauc plots obtained from absorbance measurements reported in Fig. 1, for the 0.2 µm thin films at 0 week (black circles) and after 4 weeks (black squares); (right) pump-probe transient transmittivity, ΔT/T (red), and reflectivity, ΔR/R (black), for the sample with thickness of 0.2 µm, measured at 0 week (open symbols) and after 4 weeks (solid lines).

Figure 3 (left) shows Tauc plots, obtained from the absorbance curves reported in Fig. 1, to evaluate the optical gap of the samples before and 3 week after SSSP. The absorbance coefficient α has been calculated from the curve in Fig. 1, and the expression (αhν)^1/2^ as a function of energy has been estimated. Changes in the optical gap from 2.2 eV to 1.6 eV were observed. The energy values obtained from the Tauc plots are in agreement with the excitation energy of 2.5 eV reported in literature^17, 18^ related to 6-hydroxy-4-dihydro-indol-5-one (HHI) structure and a metastable state called open rotamer of HHI keto open form (1.56 eV), and confirm that oxidative processes accompanying SSSP and aging change both electronic and structural features of the polymeric chains. This conclusion holds true also for the other samples, whereas, for the eumelanin samples prepared by AISSP, the Tauc plots at t = 0 and t = 3 weeks indicate the same value of optical energy gap (≈1.5 eV).

To confirm the presence of the band gap at about 1.5 eV in the oxidized samples, degenerate pump-probe^19–22^ measurements have been carried out (Fig. 3 (right)) at wavelength of 795 nm (1.5 eV), where the absorption of the samples is minimum and the photon energy corresponds to the energy gap estimated from the Tauc plots.

Starting from the DHI phase, in which the band gap is about 2.6 eV, use of a wavelength of 790 nm allows to measure any variation of transient transmittivity/reflectivity (∆T/T and ∆R/R), since at photon energies below the bandgap value (1.5 eV) the samples do not absorb at all. In order to monitor the change in the sample optical bandgap caused by spontaneous aerial polymerization of DHI (during the four weeks), the degenerate configuration (pump and probe at the same wavelength) would allow to achieve selectivity in detecting the energy bandgap produced by ground-state bleaching.

In Fig. 3 (right) the pump-probe response shows that in the just grown samples (open symbols) no change is observed both in time resolved reflectivity and transmittivity. At this excitation energy the sample does not absorb, meaning that the energy band gap is larger than 1.5 eV. On the other hand, the polymerization/oxidation of the sample occurred after 4 weeks from its production gives rise to an evident ground-state bleaching, testified by an increased probe beam transmission subsequent to pump excitation. This result demonstrates that the energy band gap of the oxidized samples is changed to about 1.5 eV, with a modification of the optical and electronic features of the samples.

The detection of the time resolved reflectivity/transmittivity signal that happens after 4 weeks in all the samples is related to the increased absorbance in the visible-IR range already reported in Fig. 1 (right). Moreover, the rapid (few ps) non radiative relaxation of the signal confirms that the optical excitation relaxes via a large number of energy relaxation pathways^23, 24^, which represents one of the most important feature of eumelanin for its applications in optoelectronic technology. The change in the pump-probe response could be explained by considering the broad absorption spectrum of eumelanin as resulting from a large number of heterogeneous chromophores^23^. Only those chromophores having strong absorption at the pump wavelength are excited in the pump-probe experiments, and the ground state bleaching intensifies at probe wavelength close to the pump wavelength^23^. Then, chromophore size defines the absorption: smaller chromophores account for the strong UV-vis absorption, whereas larger chromophores, with extensive electron delocalization and red-shift, account for the near-IR absorption tail^25^. This implies that longer pump wavelength would access broader absorbing chromophores, and the probe response would be increasingly dominated by ground state bleaching.

In a final set of experiments the modification of the morphological properties of the films caused by polymerization was investigated by atomic force microscopy (AFM) analysis. As mentioned above, thickness dependent changes in the normalized spectra of eumelanin films have already been reported^26^. In the previous study, however, the films were prepared from dissolution/dispersion of preformed commercial eumelan in, where by the increase in the absorption coefficient in the long-wavelength region was attributed to Mie scattering dominating in the higher energy part and increasing with film thickness.

Conversely, in the present study thickness would specifically reflect features of the polymerization process under *homogeneous solid state* conditions, and would not reflect the features of preformed eumelanin particles.

**Figure 4:**
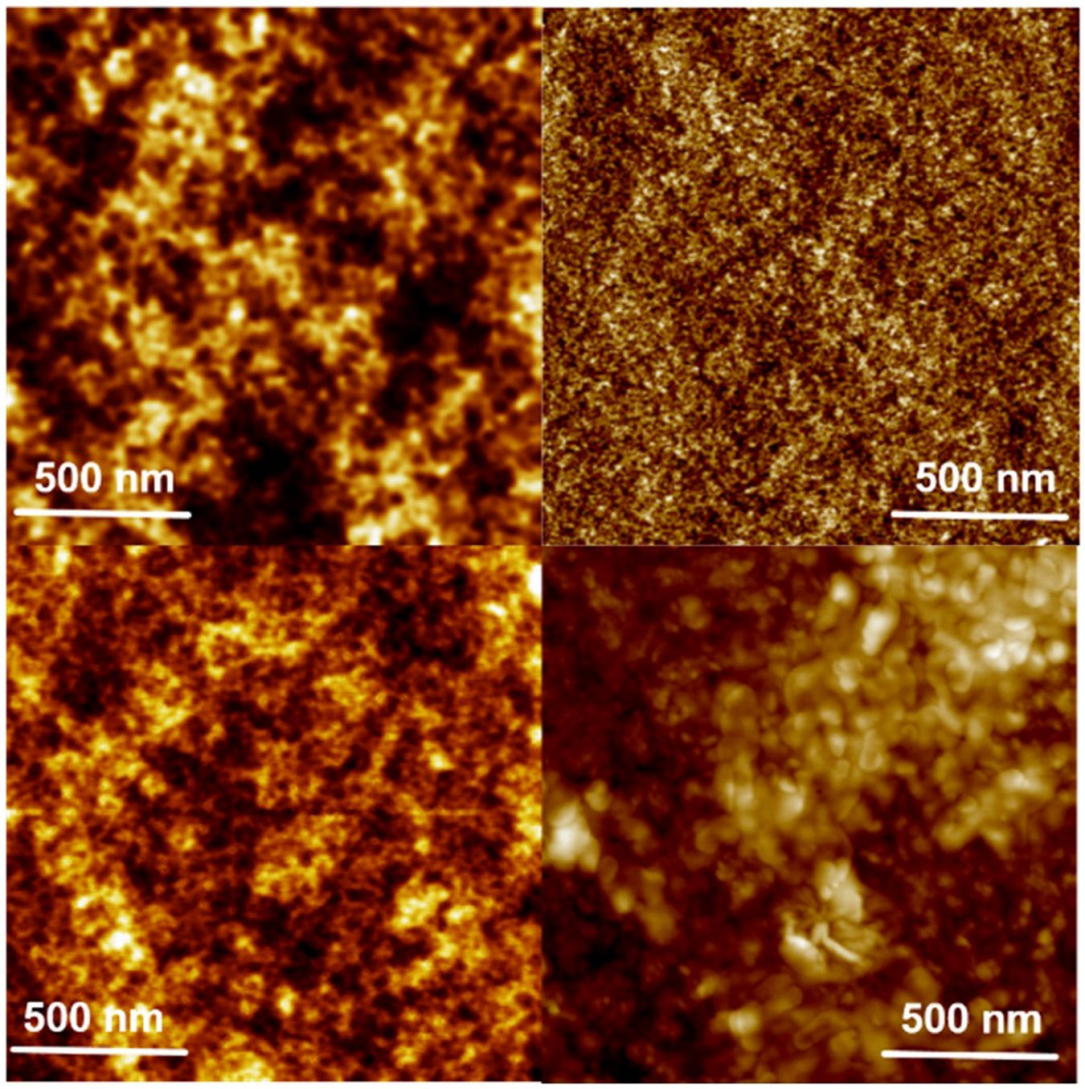
AFM topographic images of DHI thin films with thickness of 0.2 μm (upper row) and 0.5 μm (lower row) as prepared (left column) and after SSSP over 4 weeks (right column).

Figure 4 shows AFM images of DHI thin films with thickness of 0.2 μm and 0.5 μm as prepared (left) and after SSSP over 4 weeks (right). The data show significant age-dependent modifications of the films which reflect thickness-related differences in the mode of structuring of the surface upon monomer oxidation and melanin formation.

Soon after their preparation (Fig. 4-left column) the surfaces of 0.2 and 0.5 μm thick films look very similar. It is worth noting that in the thicker film (Fig. 4-bottom line), SSSP process results in a modest alteration of film surface toward formation of a fine, sand-like structure. However, polymerization leads to a coarse-grained morphology which could testify the formation of compact substructures presenting a larger absorption coefficient. Such apicture is clearly illustrated in the 3D profiles of 0.2, 0.5 and 1 μm thick films reported in SI in Figures S2 and S3﻿ and summarized in Table S1.

The elucidation of the mode of packing of DHI molecules on the substrate and, especially, of the specific changes in film structure and spatial organization at molecular level following oxidative polymerization is a difficult task in such a complex and disordered material as eumelanin, and deserves further dedicated efforts. Nonetheless, important insights can derive from a critical analysis of the present results. To put the discussion into perspective, some key concepts in eumelanin chemistry and physics, along with a critical comparison of the two preparation procedures used, need to be briefly addressed. As mentioned earlier, the only difference between the AISSP and the SSSP procedures is in the omission in the latter of the final step involving exposure of the film to gaseous ammonia.

**Figure 5:**
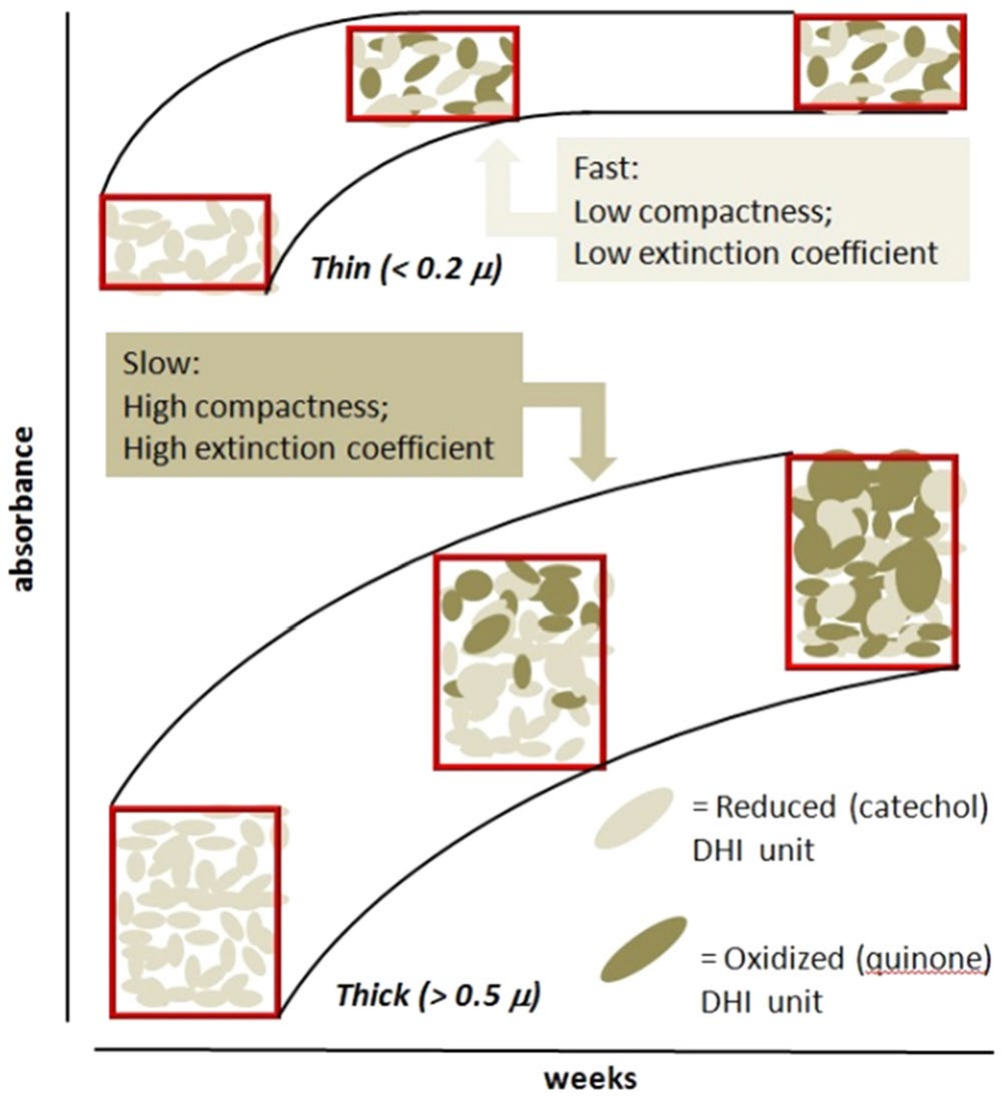
Pictorial view of DHI-unit oxidation and reorganization within melanising films of different thickness. Absorbance variation with time is associated whit the progress of unit oxidation in the film.

Since the auto-oxidative polymerization of DHI to eumelanin is accelerated by an alkaline medium causing partial deprotonation of the catechol function in DHI, and favoring electron transfer to molecular oxygen, eumelanin formation in thin films occurs at much faster rates under the AISSP conditions relative to SSSP. This difference is shown herein to have a considerable impact on the mode of reorganization of the molecular components/aggregates *in the solid state*, filling a major gap in melanin chemistry of both biological and technological relevance. It can be reasoned that the slower kinetics of DHI conversion to eumelanin in the SSSP regime may allow for gradual and more uniform packing interactions among the growing oligomer components. It is worth noting in this connection that polymerization processes are expected to decrease film volume in consequence of increasing covalent bond formation. Conversely, the faster rates of DHI polymerization that characterize the AISSP conditions would not be compatible with an efficient molecular reorganization and packing under the constraints posed by the solid state conditions, leading to spaced aggregates, due to poor packing, and a lower degree of stacking interactions affecting absorbance (see Fig. 5)^27^.

This view is supported by AFM images which evidenced correlation between morphology changes and thickness of the film as long as the conversion of DHI to eumelanin proceeds. Thus, slower kinetics lead to coarse locally compact structures purportedly accounting for larger absorption coefficients, whereas in faster processes the rate of chemical polymerization processes exceeds that of physical molecular re-organization resulting in a finely structured morphology with opposite impact on light extinction coefficients (Fig. 4, see also AFM images in SI in Figures S2 and S3).

Lastly, Raman spectra were collected via a confocal space-resolved equipment to gain insight on molecular and supramolecular structures of the investigated melanin films. Literature data provide evidence of possible Raman-based discrimination among different types of carbon-based black pigments^28^ and specifically melanins^29, 30^. Moreover the determination of particle size within samples has also been reported^31^.

Raman spectra of the 0.2, 0.5 and 1 µm thick films (see SI Figure S4) indicate that SSSP-generated melanin films of different thickness do not differ significantly in their chemical features, all sharing the characteristics G- and D-bands (at about 1590–1605 cm^−1^ and 1350–1380 cm^−1^, respectively) associated to the polymeric aromatic scaffolds^28^. Moreover a shoulder around 1200 cm^−1^ is also present, further confirming the eumelanin signature^29^.

Interestingly, normalization of the spectra at the baseline revealed little, but significant, differences in the signal intensities associated with the film thickness, as thicker films featured a more intense signal. Although several factors can affect the signal intensity of Raman spectra, including diffraction index, it may be speculated that the signal intensity increase observed in thicker films is associated to higher inelastic back scattering as a signature of denser material^31^.

## Conclusions

In conclusion, we have disclosed herein the SSSP procedure on DHI thin films as a simple and ingenuous means of inquiring into the origin of eumelanin broadband absorption in the solid state. The results demonstrate, for the first time to the best of our knowledge, an anomalous evolution of optical properties depending on both kinetics of DHI polymerization and film thickness. This observation would reflect a complex interplay of physical and chemical processes during eumelanin formation in the solid state dictated by a more or less profound dynamic molecular reorganization process with alteration of the various levels of disorder. Although scattered observations on the thickness-dependence of eumelanin absorption properties in thin films can be found in the literature^32^, the use of chemically ill-defined commercial melanins and/or harsh deposition conditions and the limited characterization of the phenomenon prevented a clear-cut rationalization and correlation with structural features. The present SSSP approach benefits from the highest possible level of control over molecular mobility and is amenable to several levels of modification for enhancing and tuning absorption properties of eumelanin films. Besides the general interest related to the origin of eumelanin broadband absorption, the results of this study offer a novel potentially versatile tailoring strategy which has applicability for the design of eumelanin-based optoelectronic materials.

## Methods

All absorbance spectra of eumelanin and DHI samples have been recorded with a commercial spectrophotometer (Perkin-Elmer, mod. Lambda 900). It is worth mentioning here that the contribution of the glass substrate sustaining sample is automatically subtracted from the acquired spectra by inserting a second identical substrate without any deposition into a reference arm of the spectrophotometer. Moreover, interference fringes occurring at wavelengths close to the film thickness have been averaged out with a software smoothing procedure.

Time-resolved spectroscopic measurements were performed using a standard degenerate pump–probe technique, based on a mode-locked Ti:Sapphirelaser oscillator, delivering pulses at 82 MHz repetition rate, with 790 nm center wavelength and 100 fs duration. The photo-induced transmittivity and reflectivity change were measured using a pump fluence of about 30 µJ/cm^2^ and a weaker probe pulse (1:30 ratio of probe and pump beam power). The two beams were cross-polarized (with the probe beam in the s-polarization state) to prevent the detected signal from coherent artifact. The focal spot diameter of the pump beam is 60 μm, approximately three times larger than the probe beam spot size, in order to guarantee a nearly uniform excitation region within the probed area. The reflectivity and transmittivity variation (ΔR and ΔT) have been normalized to the DC value of the (reflected and transmitted, respectively) probe signal, which is not affected by the pump signal. By convention, we set the lock-in amplifier phase so that a negative (positive) signal of the ΔR/R (ΔT/T) indicates a ground state-bleaching. Each scan reported in Fig. 3 (right) is the average of 10 acquisitions. Further experimental details can be found in refs 19, 20 and 21.

More detailed experimental procedures are reported in the SI.

## Electronic supplementary material

Supplementary informations
