# Supplementary material for: Anomalous evolution of broadband optical absorption reveals dynamic solid state reorganization during eumelanin build-up in thin films
Source: Sci Rep. 2017 Mar 31;7:522. doi: 10.1038/s41598-017-00597-8 (PMC5428701; doi:10.1038/s41598-017-00597-8)
Supplement: Supplementary file 1 — Supplementary informations [file 41598_2017_597_MOESM1_ESM.pdf]

Anomalous evolution of broadband optical absorption reveals dynamic solid state reorganization during eumelaninbuildup in thin films

Carmela Bonavolontà,*[1,2] Corrado de Lisio,[1,2,3] Marco d'Ischia,[4] Pasqualino Maddalena,[1] Paola Manini,[4] Alessandro Pezzella,*[2,4,5] Massimo Valentino,[2,3]

1-Department of Physical Sciences“E. Pancini”, University of Naples “Federico II” Via Cintia 4, I-80126 Naples, ItalyE-mail:bonavolo@na.infn.it

2-INFN, Sezione di Napoli, Via Cintia 2, 80126 Napoli, Italy

3-CNR-SPIN U.O.S. di Napoli, Via Cintia 2, 80126 Napoli, Italy

4- Department of Chemical Sciences, University of Naples “Federico II” Via Cintia 4, I-80126 Naples, Italy

5 - Institute for Polymers, Composites and Biomaterials (IPCB), CNR, Via Campi Flegrei 34, 80078 Pozzuoli (Na), Italy E-mail: alessandro.pezzella@unina.it

Absorption profiles of DHI and eumelanin films 1S

AFM 3D topographic images of DHI and eumelanin films (0.2 and 0.5 μm) 2S

Roughness Parameters 3S

AFM profiles (2D and 3D) of eumelanin films 0.2 μm 4S

AFM profiles (2D and 3D) of eumelanin films 0.5 μm 5S

AFM profiles (2D and 3D) of eumelanin films 1 μm 6S

RAMAN spectra of 0.2, 0.5 and 1 μm eumelanin films 7S

UV profile of DHI films before and after accelerated aging 8S

Methods 9S

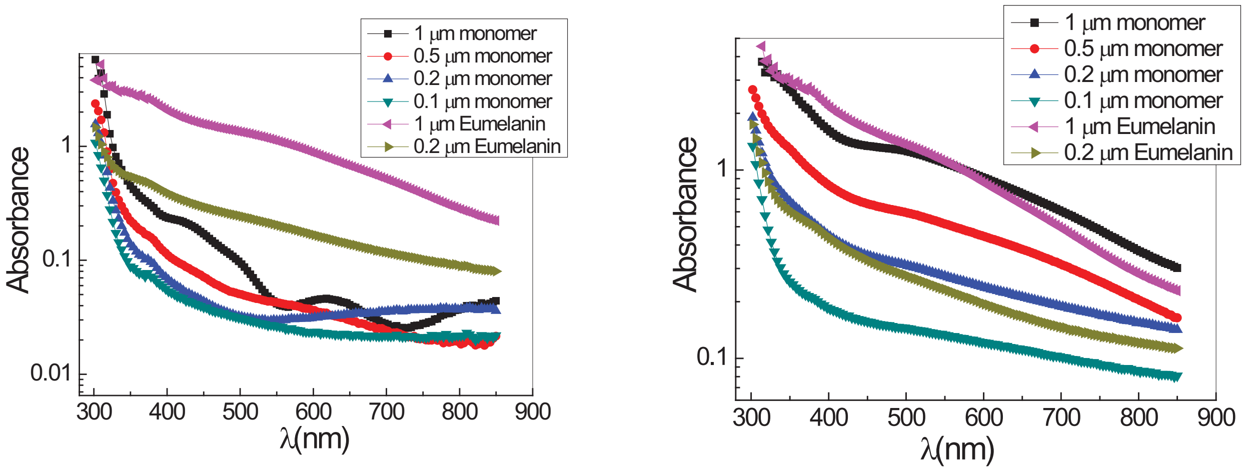

Figure S1. Absorption spectrum of DHI (left) just after deposition (0 week), and (right) after aging (4 weeks). For comparison the eumelanin thin film 0.2 um and 1um prepared by the AISSP at 0week and after 4 weeks are also reported.

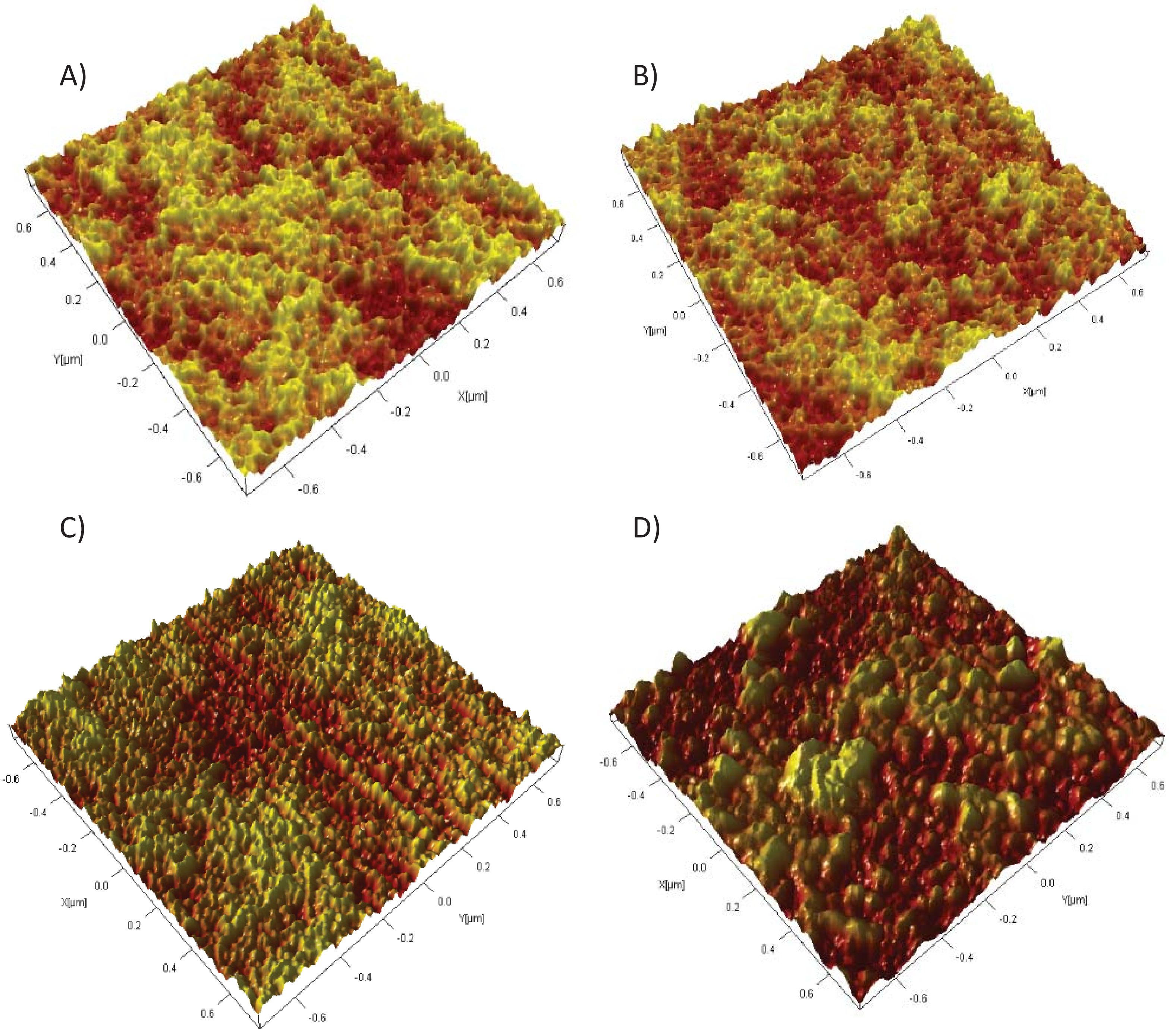

Figure S2. AFM 3D topographic image 1,5x1,5 µm: (a), (c) the DHI and SSSP samples with thickness of 0,2 µm, respectively; (b) and (d) the DHI and SSSP (after 4weeks) samples with thickness of 0,5 µm, respectively. The color scale ranging from 1 nm to 22 nm along the Z-axis (see table S1).

	Sample (a) DHI 0.2 µm	Sample (c) SSSP 0.2 µm	Sample (b) DHI 0.5 µm	Sample (d) SSSP 0.5 µm
**Roughness average**	**0,29 nm**	**0,32 nm**	**0,23 nm**	**3,62 nm**
**Root Mean Square**	**0,37 nm**	**0,40 nm**	**0,29 nm**	**4,64 nm**
**Max Valley Depth**	**1,28 nm**	**1,63 nm**	**1,17 nm**	**14,57 nm**
**Max Peak Height**	**1,50 nm**	**1,72 nm**	**1,17 nm**	**21,38 nm**

Table S1: Values of Roughness average, Root Mean Square, Max Valley Depth and Max Peak Height, obtained from the AFM image shown in figure S1 and in the Figure 3 of the main text.

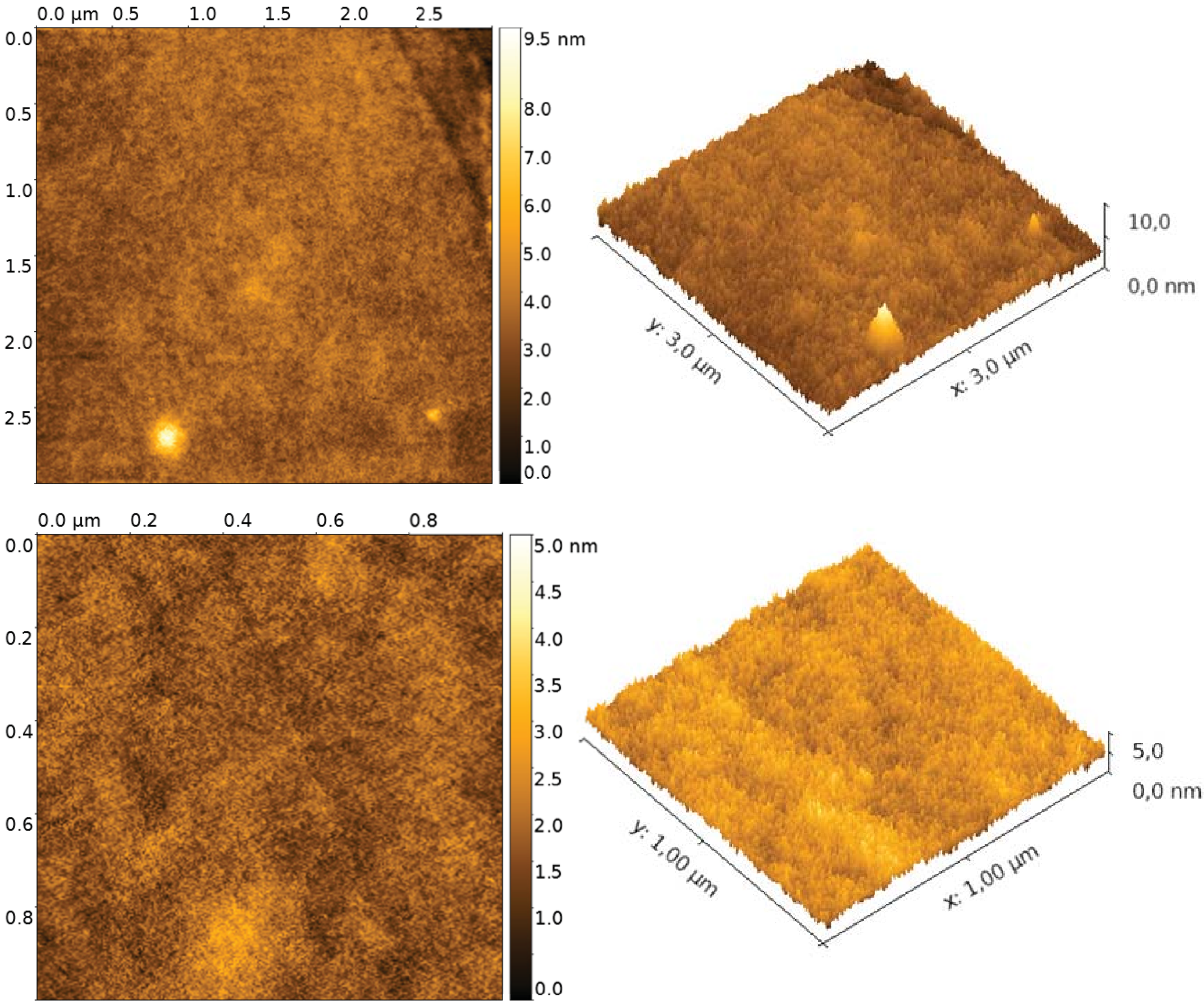

Figure S3. AFM 2D e 3D topographic images of sample with thickness of 0.2 μm after SSSP over 4 weeks.

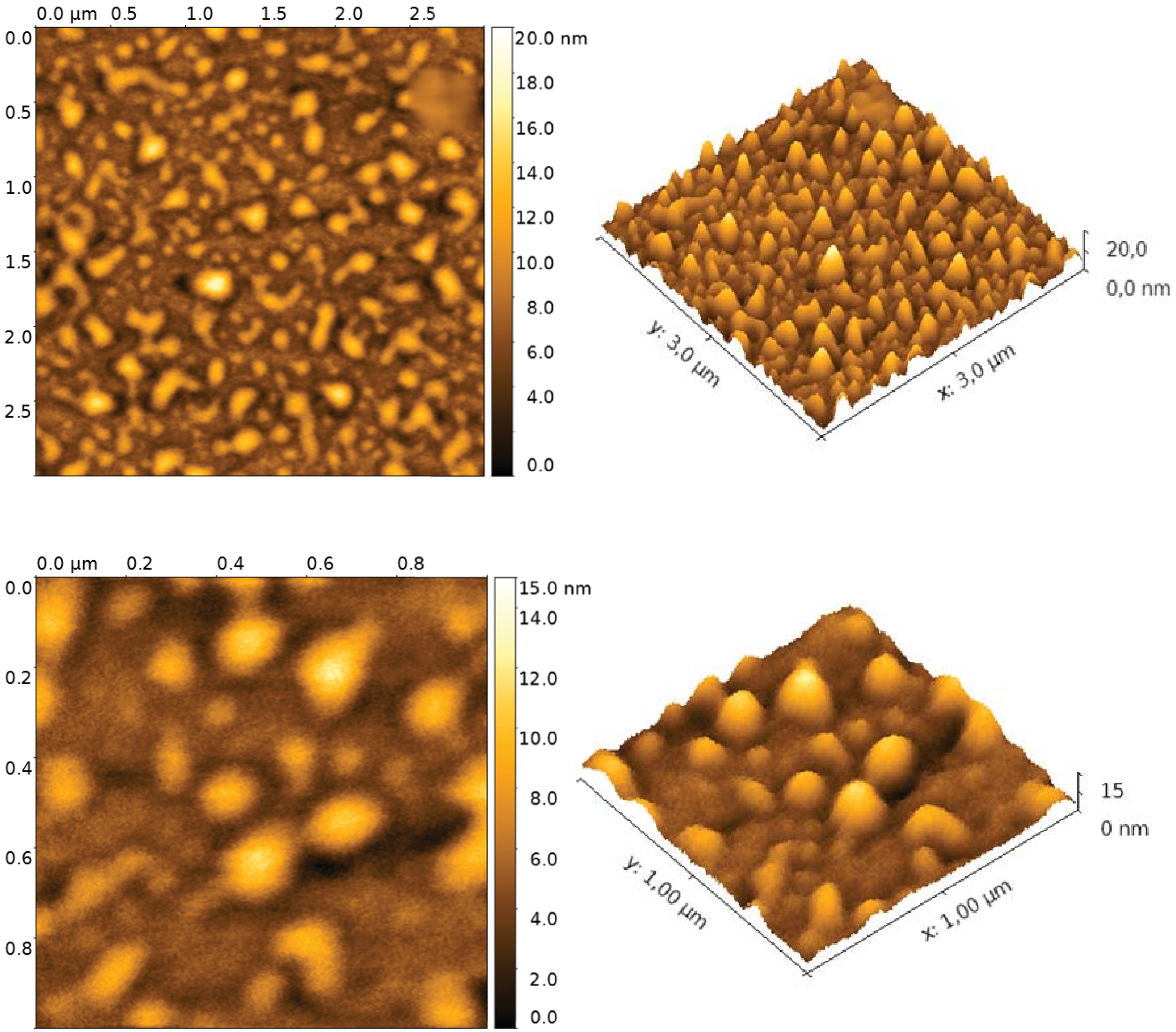

Figure S3. AFM 2D e 3D topographic images of sample with thickness of 0.5 µm after SSSP over 4 weeks.

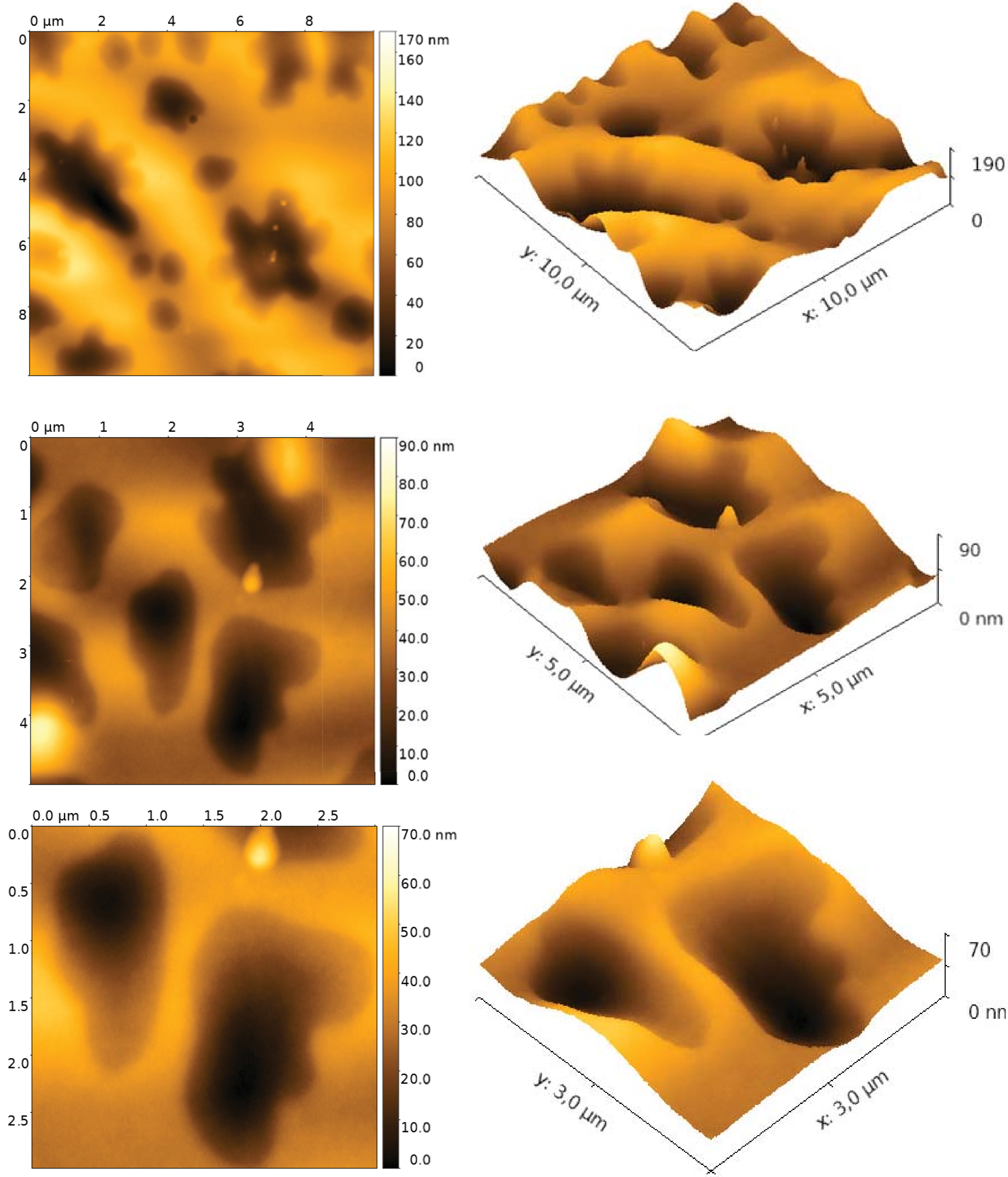

Figure S3. AFM 2D e 3D topographic images of sample with thickness of 1 µm after SSSP over 4 weeks

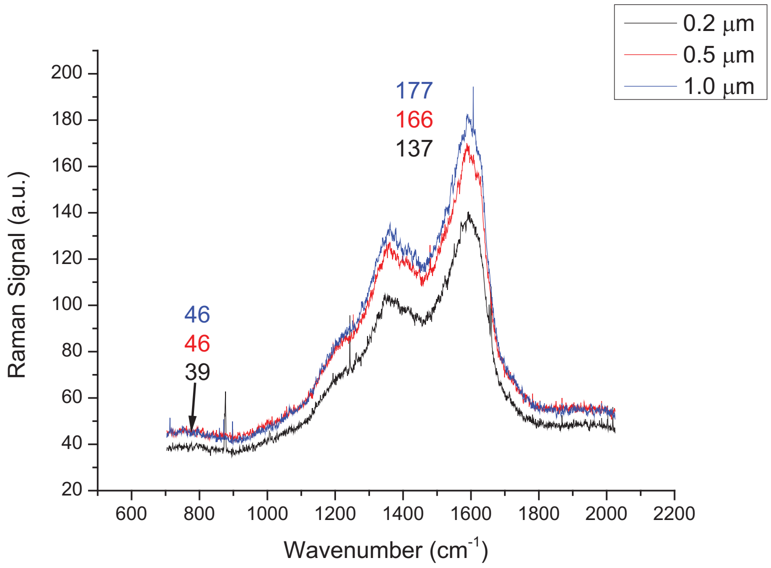

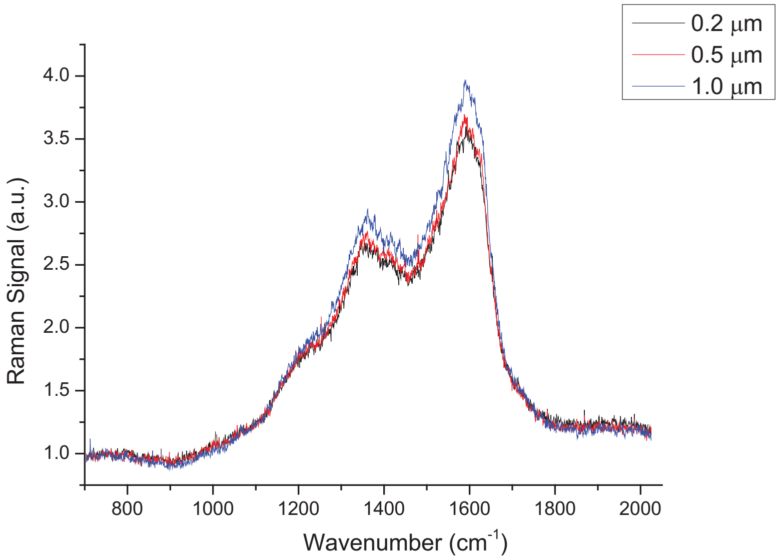

Figure S4. RAMAN spectra of 0.2, 0.5 and 1 μm of of DHI films after 4 weeks SSSP (upper plot) and the same spectra after baseline count correction was applied subtraction 46, 46 and 39 counts (lower plot).

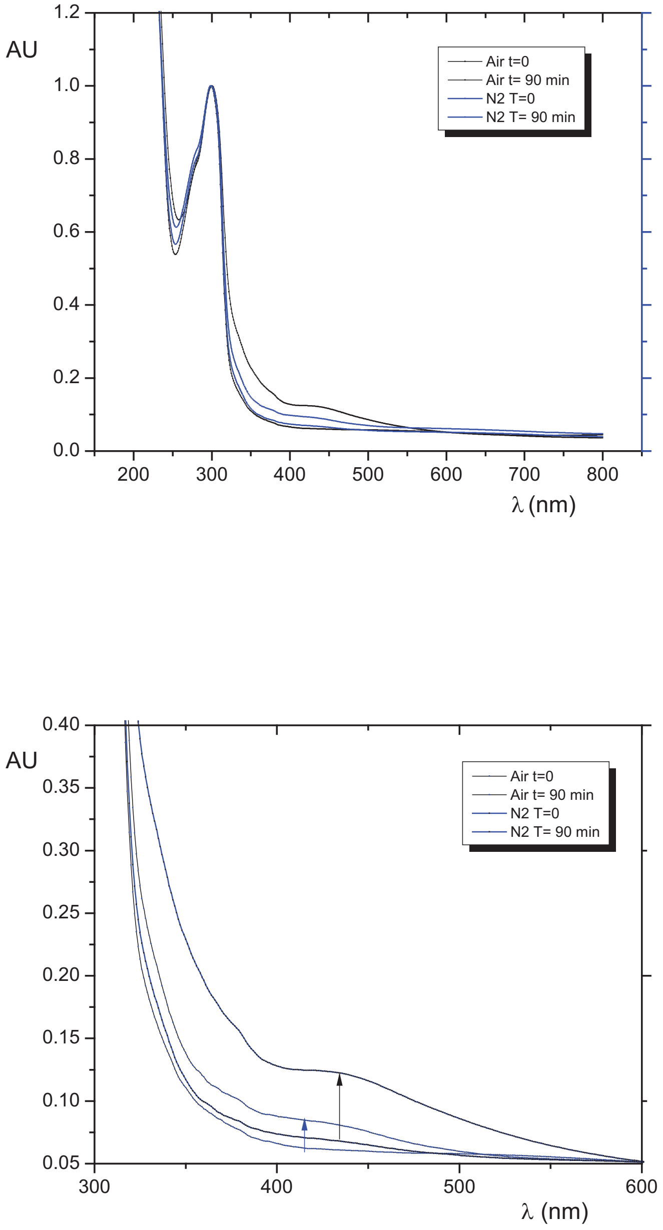

Figure S5. UV profiles of DHI films before and after 90 min accelerated aging at 60 °C in N_2 (bleu lines and air (black lines). In the zoomed region of the lower plot arrows denote the trend (N_2 bleu, air black arrows).

Methods

Film Morphology

Film thickness was estimated by scratching the film down to the substrate with a needle and measuring by means of AFM the height of the resulting trench (XE100 Park instrument operating in non-contact mode amplitude modulation, silicon nitride cantilever from Nanosensor at room temperature in ambient conditions).

Atomic Force Microscopy images have been recorded by a commercial high-resolution AFM Systems Powered by PeakForce Tapping provided by Bruker. The high-resolution Atomic imaging of the samples is performed in tapping mode on a scanning area of about 1.5 µm x 1.5 µm, at room temperature. The topographic signal is reported after the image processing obtained using the Nanoscope Analysis software.

RAMAN and AFM characterization.

The topography of the samples was investigated by an Atomic Force Microscope (AFM – Witec Alpha 300 RAS) working in tapping mode with non-contact cantilevers (resonance frequency of 280kHz and spring constants of 42N/m). This operation mode is well suited for imaging soft samples.

Raman characterization was performed through a confocal optical microscope using a 488 nm laser excitation wavelength(5 mW power) and a high numerical aperture microscope objective(50x magnification, 0.75 NA). The signal is collected by an optical fiber (50 µm core which acts as confocal pinhole) and sent to a 300mm focal length spectrometer equipped with a thermo-electrically cooled, back-illuminated CCD detector.

Several Raman spectra were acquired in different positions for each sample in order to take into account variations due to local inhomogeneity of the samples. The spectra were averaged and their mean value is shown.

DHI Thin Film Deposition:

DHI thin films were prepared by spin coating with a Laurell WS-650MZ-23NPP/LITE coater; thin films were deposited on quartz or glass substrates. Thin films were obtained from a 30 mg/mL solutions of DHI in methanol after filtering through a 0.2 µm nylon membrane, using the following speed gradients: 2000 rpm for 90”; 800 rpm for 10” and 3000 rpm for 60”; 2000 rpm for 60”; and 3000 rpm for 90”. In some cases thin films were annealed at 70 °C for 30’ under a nitrogen atmosphere.

Ammonia-Induced Solid State Polymerization:

The oxidation of DHI thin films (100-200 nm thickness) to give the melanin polymer has been achieved by exposure to an oxidizing atmosphere (e.g. oxygen atmosphere and ammonia vapors). In the general procedure, the appropriate film was incubated in the oxygen/ammonia atmosphere at controlled temperature (25 - 40 °C). The ammonia vapors were produced by equilibration of the atmosphere with ammonia solution (28% to 7% NH3 in H_2O) in a sealed camera at 1 atm pressure. Exposure times varied in the 2 - 18 h range.
